# Encapsulating peritoneal sclerosis: from early diagnosis to successful kidney transplantation

**DOI:** 10.1590/2175-8239-JBN-2021-0001

**Published:** 2021-04-23

**Authors:** Marina Reis, Ana Marta Gomes, Clara Santos, Daniela Lopes, João Carlos Fernandes

**Affiliations:** 1 Centro Hospitalar Vila Nova de Gaia/Espinho, Departamento de Nefrologia, Vila Nova de Gaia, Portugal.

**Keywords:** Peritoneal Dialysis, Peritoneal Fibrosis, Kidney Transplantation, Tamoxifen, Diálise Peritoneal, Fibrose Peritoneal, Transplante de Rim, Tamoxifeno

## Abstract

Encapsulating peritoneal sclerosis is an uncommon but serious complication of peritoneal dialysis. In most cases, the symptoms appear after peritoneal dialysis withdrawal, which hampers its diagnosis. We present the case of a 44-years-old Caucasian male who had been on peritoneal dialysis for 6 years and 3 months and was switched to hemodialysis due to ultrafiltration failure. During his last months on peritoneal dialysis, he developed anorexia and asthenia, which were initially attributed to dialysis inadequacy. After hemodialysis induction, the patient developed abdominal pain, increased abdominal volume, obstipation alternating with diarrhea, and weight loss. Computed tomography showed de novo ascites. A diagnosis of early encapsulating peritoneal sclerosis was considered, and treatment was promptly initiated with nutritional support, oral prednisolone, and tamoxifen for one year. The patient progressed with resolution of the symptoms. One month after the end of the treatment, he underwent a successful kidney transplant and remain without any major intercurrences. A high level of clinical suspicion is crucial for the early diagnosis of encapsulating peritoneal sclerosis as the disease can be fatal in advanced stages. This case highlights that with early treatment, kidney transplantation can be successfully performed after an episode of encapsulating peritoneal sclerosis.

## Resumo

A esclerose peritoneal encapsulante é uma complicação incomum, mas grave, da diálise peritoneal. Na maioria dos casos, os sintomas aparecem após a suspensão da diálise peritoneal, o que dificulta seu diagnóstico. Apresentamos o caso de um homem caucasiano de 44 anos de idade que esteve em diálise peritoneal por 6 anos e 3 meses e foi transferido para hemodiálise devido a falha de ultrafiltração. Durante seus últimos meses em diálise peritoneal, ele desenvolveu anorexia e astenia, que foram inicialmente atribuídas à inadequação da diálise. Após a indução de hemodiálise, o paciente desenvolveu dor abdominal, aumento do volume abdominal, obstipação alternada com diarreia, e perda de peso. A tomografia computadorizada mostrou ascite de novo. Foi considerado um diagnóstico de esclerose peritoneal encapsulante precoce, e o tratamento foi prontamente iniciado com suporte nutricional, prednisolona oral e tamoxifeno por um ano. O paciente progrediu com resolução dos sintomas. Um mês após o término do tratamento, ele foi submetido a um transplante renal bem-sucedido e permanece sem maiores intercorrências. Um alto nível de suspeita clínica é crucial para o diagnóstico precoce da esclerose peritoneal encapsulante, uma vez que a doença pode ser fatal em estágios avançados. Este caso destaca que, com tratamento precoce, o transplante renal pode ser realizado com sucesso após um episódio de esclerose peritoneal encapsulante.

## Introduction

Encapsulating sclerosing peritonitis (EPS) is a serious complication of peritoneal dialysis (PD) caused by an inflammatory process that diffusely affects the peritoneum, resulting in fibrosis that encapsulates the small intestine and leads to obstruction^1^^-^ 3. EPS is rare, and the reported incidence is between 0.7 and 3.3%^1^. High morbidity and mortality occur due to bowel obstruction and malnutrition. The mortality rate is reported to be around 50% and usually occurs within 12 months after the diagnosis^2^^,^ 3. In 70-90% of the cases, EPS is diagnosed after withholding PD. The mechanisms involved in the development of EPS are complex and include inflammation and the dysregulation of growth factors, particularly vascular endothelial growth factor (VEGF) and transforming growth factor-β (TGF-β). It is believed that glucose degradation products contained in the dialysate and multiple peritonitis episodes may play a role in peritoneal inflammation and neoangiogenesis, which ultimately lead to peritoneal fibrosis^3^^-^ 6. The symptoms of EPS are unspecific, which makes its clinical diagnosis challenging. On the initial inflammatory stage, early satiety, loss of appetite, nausea, diarrhea, weight loss, and abdominal pain could be the only symptoms. Less often, signs of EPS begin when the patient is still on PD with bloody peritoneal effluent, ineffective ultrafiltration, and transition to high peritoneal transporter status. Laboratory parameters, at this stage, may show hypoalbuminemia and increased inflammatory markers^3^. Abdominal computer tomography (CT) is the best imaging exam, although it has low sensitivity because CT alterations are unspecific and only have value in the context of clinical suspicion. Moreover, in the early inflammatory phase, abdominal CT may not show any bowel alteration besides ascites. Late symptoms are intermittent subacute bowel obstruction (fibrous stage). At this point, peritoneal thickening can be recognized by peritoneal enhancement after CT intravenous contrast, as well as peritoneal calcification, loculated fluid collections, tethering of the small intestine, and intestine wall thickening^3^^-^ 5. Definitive diagnosis is obtained by peritoneal biopsy^3^.

Early treatment, before late-stage fibrosis, is more effective, so awareness of this diagnosis is crucial for successful outcomes^7^^-^ 9. Though there is lack of evidence regarding the best medical treatment options, a regimen with glucocorticoids and tamoxifen is the most consensual^9^^-^ 11. Kidney transplantation remains a valuable option after EPS treatment, and with that in mind, patients should be referred to a transplantation center early after EPS resolution^2^^,^ 8 ^,^ 9.

## Case Presentation

A 44-years-old Caucasian male with stage 5 chronic kidney disease had been on continuous ambulatory PD for 5 years without any episodes of peritonitis during that time. After this, he developed hypervolemia and was switched to automated PD and higher glucose-containing peritoneal solutions were used to increase the ultrafiltration. A modified peritoneal equilibration test (mPET) was performed, which revealed a transition from high transporter (dialysate to plasma (D/P) creatinine 0.83) to average transporter (D/P creatinine 0.65). Unfortunately, free water transport was not available due to an error in PD fluid sampling. The patient also developed symptoms of anorexia, hypoalbuminemia, despite the effectiveness of creatinine and urea removal according to routine measurements (Table 1).

**Table 1:** Results of last two analytical studies on peritoneal dialysis

	26/03/2018	21/05/2018
Hemoglobin, g/L	11.3	10.8
Ferritin, ng/mL	285	394
Urea, mg/dL	119	132
Creatinine mg/dL	14.39	15.36
Ultrafiltration (24H), mL	1145	1575
Renal KtV	0	0
Peritoneal Kt/V	1.79	1.90
nPCR	0.9	1.0
Sodium, mmol/L	133	137
Potassium, mmol/L	5.95	5.24
Corrected Calcium, mg/L	10.46	10.06
Phosphorus, mg/dL	3.8	4.5

nPCR: Normalized protein catabolic rate; PTH: parathyroid hormone.

**Figure 1:**
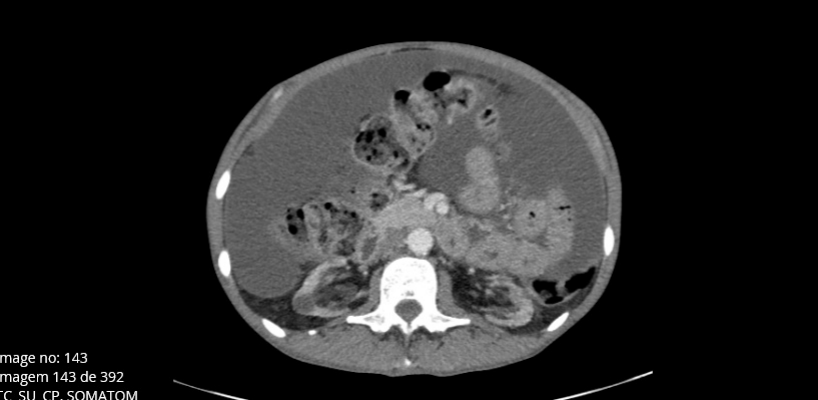
Ascites in abdominal CT raised the suspicion for encapsulating peritoneal sclerosis

The patient then had his first peritonitis episode, which was caused by *Streptococcus salivarius,* and a repeat episode occurred two months later. At this point, he switched to nocturnal HD (six hours per session, thrice weekly). The symptoms of anorexia and asthenia remained, and the patient developed abdominal pain, increased abdominal volume, obstipation alternating with diarrhea, and unintended weight loss (4 kilograms in 4 months). Blood analysis showed a C-reactive protein level of 7 mg/dL, hypoalbuminemia at 3 mg/dL, and anemia (hemoglobin 9.4 g/dL). The imaging study showed de novo ascites in abdominal echography and abdominal CT without significant bowel alterations (Figure 1). The patient had no signs of hypervolemia, and the ascites remained despite increased ultrafiltration.

Diagnostic paracentesis was performed, and the peritoneal fluid was an exudate with bloody appearance, increased leukocytes (200/uL), predominant mononuclear cells (99.5%), raised erythrocytes (2000 /uL), proteins at 4.9 g/dL, LDH at 130 U/L, and glucose at 81 mg/dL. Cultures were negative, including for aerobic bacteria, fungal, and mycobacteria, and the cytology result was negative for malignant cells. A colonoscopy did not show significant alterations, and the results for the fecal occult blood test, *Giardia lamblia,* and *Cryptosporidium* were negative.

Although there was no obvious imaging sign of peritoneal sclerosis, early encapsulating peritonitis was suspected based on the clinical presentation (malaise, anorexia, weight loss, and abdominal pain), elevated inflammatory markers, and *de novo* ascites in a patient who was on PD for long period. Treatment was promptly initiated with oral nutritional support and tamoxifen 20 mg every 12 hours for one year. During the first month, 1 mg/kg/day of prednisolone was given, and then slowly tapered until 10 mg, at six months, and then continued for one year.

Two months after the initiation of EPS treatment, the patient presented complete resolution of the symptoms and regularization of inflammatory markers. Abdominal CT at 6 months showed complete ascites resolution and excluded other signs related to EPS development. One month after the end of EPS treatment, it was possible to perform a kidney transplant successfully, and the transplant has remained for almost two years thus far without any significant complications.

## Discussion

In this case, EPS was diagnosed after PD withdrawal, which is the most common timing for presentation. It is hypothesized that after PD cessation, inflammatory reaction persists, and deposited fibrin is no longer removed by PD exchanges, which leads to fibrosis formation^6^. The greatest EPS risk factor is length of time on PD, mostly for more than five years. Other factors that may lead to dysregulated peritoneal inflammation are high dialysate glucose exposure, the use of conventional PD solutions (as opposed to biocompatible PD solutions), severe or frequent peritonitis episodes (especially by *Staphylococcus aureus, Pseudomonas* spp*.*, and fungi), younger age, abdominal surgery, β-blocker use, and higher peritoneal solute transport status^8^^,^ 12 ^,^ 13. The most important risk factors in this case were the length of time on PD and the higher peritoneal solute transport status.

In our patient, symptoms were highly unspecific and were initially attributed to PD inadequacy. In his last year on PD, the patient developed volemia control issues that were initially attributed to a loss of residual renal function. However, there was little improvement after switching to automated PD and higher glucose-containing peritoneal use to improve ultrafiltration. mPET showed ultrafiltration failure and a transition from high peritoneal transporter to average transporter. In retrospect, these could have been signs of early EPS.

After exclusion of gastrointestinal pathology and with the diagnosis of de novo ascites (although without bowel alterations), elevated inflammatory parameters, and hypoalbuminemia, EPS was suspected, and early treatment was instituted. The diagnosis of EPS in this early inflammatory state is challenging. Those alterations are highly unspecific and sometimes not immediately associated to EPS, since most of the literature and case reports focus on more severe obstructive symptoms that occur late in the course of the disease^14^^-^ 16.

There is lack of evidence regarding EPS best management. Treatment options include nutritional support, drug therapy, and surgery in refractory cases as well as other more controversial treatments such as regular peritoneal lavage^8^^-^ 11 ^,^ 17 ^,^ 18.

In this case, treatment with tamoxifen and glucocorticoids was used. A large retrospective Dutch study demonstrated reduced mortality in EPS patients treated with tamoxifen compared to patients who were not treated with tamoxifen (45.8 versus 74.4%, p=0.03)^10^. In contrast, The Pan-Thames EPS study showed no improvement in survival rate when tamoxifen was used^11^. The discrepancy in outcomes may be due to the inclusion of more severe cases in the Dutch study. After the introduction of tamoxifen therapy, favorable clinical outcomes are often seen within two to six months^8^. Corticosteroids are particularly important in the inflammatory stage. In the Dutch EPS study, the multivariate analysis with adjustment for concomitant prednisone use in the tamoxifen-treated group confirmed the trend of improved survival^10^.

Malnutrition is a frequent consequence of EPS, and nutritional support is an important component of management. Improving the nutritional status of these patients is of paramount importance as it may improve the response to conservative management or avoid subsequent surgical complications^8^^,^ 18. In this patient, it was possible to provide oral nutritional supplements with good results.

Prior diagnosis and treatment of EPS are not contraindications for kidney transplantation. EPS occurs after transplantation only in patients that have been exposed to PD for several years, so by itself, transplantation does not increase the risk of developing EPS^8^. There have been isolated reports of dramatic resolution of established EPS following renal transplantation, which is possibly the result of the use of immunosuppression^19^. In our patient, kidney transplantation was possible at one month after the conclusion of EPS treatment, and the transplant has remained for two years now without major complications.

In conclusion, EPS is a devastating complication of PD. After withdrawal from PD, a high level of clinical suspicion is crucial for early diagnosis (before the fibrous stage) and successful treatment. There is no evidence for EPS screening of long-term PD patients, so strategies to improve early diagnosis are particularly important, including patient information regarding the disease and awareness among nephrologists. Kidney transplant can be performed safely and remains a valuable option in these patients.
